# Corticomotor Responses to Attentionally Demanding Motor Performance: A Mini-Review

**DOI:** 10.3389/fpsyg.2013.00165

**Published:** 2013-04-08

**Authors:** Daniel T. Corp, Hannah G. K. Drury, Kayleigh Young, Michael Do, Tom Perkins, Alan J. Pearce

**Affiliations:** ^1^Cognitive and Exercise Neuroscience Unit, School of Psychology, Deakin UniversityMelbourne, VIC, Australia; ^2^Centre for Mental Health and Wellbeing, School of Psychology, Deakin UniversityMelbourne, VIC, Australia

**Keywords:** attention, dual task, inhibition, transcranial magnetic stimulation, motor cortex, mini-review

## Abstract

Increased attentional demand has been shown to reduce motor performance, leading to increases in accidents, particularly in elderly populations. While these deficits have been well documented behaviorally, their cortical correlates are less well known. Increased attention has been shown to affect activity in prefrontal regions of the cortex. However there have been varying results within past research investigating corticomotor regions, mediating motor performance. This mini-review initially discusses past behavioral research, before moving to studies investigating corticomotor areas in response to changes in attention. Recent dual task studies have revealed a possible decline in the ability of older, but not younger, adults to activate inhibitory processes within the motor cortex, which may be correlated with poor motor performance, and thus accidents. A reduction in cortical inhibition may be caused by neurodegeneration within prefrontal regions of the cortex with age, rendering older adults less able to allocate attention to corticomotor regions.

## Introduction

Within studies of human movement and performance, there is growing research into motor deficits occurring due to changes in attention. Indeed, a catalyst for this research is the high incidence of accidents experienced by people during gross motor tasks, with approximately 32–42% of adults aged over 70 experiencing a fall each year, and falls accounting for 40% of all injury deaths (World Health Organization, 2008). In investigating the cause of these motor deficits, past research has focused primarily on behavioral studies, which have demonstrated that tasks requiring greater cognitive resources cause deficits in functional motor outcomes such as walking variability, and balance (Pellecchia, 2005; Li et al., 2010). Consequently, investigators often employ a dual task (DT) protocol to isolate the effect of additional attention on motor performance. The DT paradigm involves the concurrent execution of two motor, cognitive, or sensory tasks, which often results in poorer performance when compared to a single task (ST). A systematic review and meta-analysis by Al-Yahya et al. (2011) examined studies that had measured gait performance with an additional cognitive task. Results demonstrated that a DT condition caused disturbed gait when compared to the ST condition, including impaired gait speed, stride length, and stride time variability. Significantly, analyses also demonstrated a strong association between age and gait disturbances. Perhaps as a result of cognitive interference, accidents caused by deficits in motor performance have been attributed to an inability of older persons to properly attend to attentionally demanding motor behavior, with additional information rendering a person less able to devote cortical resources to a particular motor task (Beauchet et al., 2007; Li et al., 2010). While behavioral studies of this kind have identified attention as a causative factor in accidents, there has been comparatively less research devoted to the underlying cortical responses during motor performance, and fewer experiments comparing old and young populations within this paradigm. This mini-review will initially present prominent theories explaining reductions in motor performance with additional attention. Research demonstrating cortical changes with attention will then be discussed, with a focus on DT methods due to their ability to isolate and control levels of attention within experiments. In doing so, this mini-review refers to articles that have used a method to investigate cortical responses to changes in attention during motor tasks. These include neuroimaging methods, such as functional magnetic resonance imaging (fMRI), or electrophysiological methods, such as transcranial magnetic stimulation (TMS). Finally, studies that have used these methods to assess older populations within a DT paradigm will be analyzed and discussed.

## Scope of Mini-Review: Selection Criteria for Reporting of Evidence

The aim of this mini-review was to present literature in relation to corticomotor responses to attentionally demanding motor performance. Consequently, the authors performed database searches using combinations of the terms: attention*, “dual-task*” or “dual task*”, concurrent*, and motor cort*. The search was refined by including peer-reviewed papers printed in English between 1995 and 2012. While these searches were conducted thoroughly, some articles found using these methods were not reported, and certain articles gathered outside this search were reported as evidence if deemed appropriate by all authors.

This mini-review targeted studies that had used motor tasks that would place demand on corticomotor areas (Brodmann areas 4 and 6), with an emphasis on DT studies that had used at least one of these motor tasks. Motor activities are broad in nature and range from tasks that are processed highly automatically, to those that require a high level of cognition. For this reason, it is almost impossible to provide an unequivocal description of what a “motor task” requires of a performer (Wood, 1986). However, given that authors aim to report activity within motor regions in response to changes in attention, of interest to this review were studies that used motor tasks that required higher levels of volitional and self-initiated movement; rather than those requiring automatic responses where movement was used in response to an external cue, such as a reaction time test (For examples, see Marois et al., 2006; Mochizuki et al., 2007).

In each of the DT studies cited within this review, the DT condition is compared to the ST condition to isolate the effect of an additional task on cortical activity. Therefore, the ST is used as the control condition, and the DT used as the experimental condition.

## Theories Underpinning Deficits in Dual Task Performance

In explaining the effect of an additional task on the cortex, there are currently two theories prevailing within DT literature. The first is known as the “bottleneck theory,” whereby humans experience interference between concurrent tasks, leading to the loss in performance efficacy (Pashler, 2001; Tombu et al., 2011). Pashler (2001) suggests that a bottleneck occurs in neural processing because both tasks require the use of a single neural pathway which, as a result, cannot cope with increased demand. Alternatively, the “limited capacity theory” proposes that the brain can perform two tasks concurrently, particularly simplistic or well learned tasks, until the complexity of tasks become too difficult, at which point the brain becomes overwhelmed resulting in performance degradation of DT (Kahneman, 1973; Lavie, 2005). Thus, a bottleneck model theorizes that during attentionally demanding motor tasks, motor areas cannot be activated optimally. And that this is due to an additional task causing interference along a neural pathway converging on the motor structures mediating these tasks.

## Dual Task Motor Performance: Evidence from Studies Investigating Cortical Activity

Increased attention to non-motor tasks has been shown to result in greater cortical activity within prefrontal regions (Courtney et al., 1997; Kastner et al., 1999). As proposed by Al-Yahya et al. (2011) motor performance also requires the use of high-order cognitive systems, with the additional task within a DT test interfering with the ability to control the motor task. As a result of this increased demand, DT studies using fMRI have shown that performance of an additional, simultaneous task results in higher activity within prefrontal areas of the cortex (Erickson et al., 2005; Poldrack et al., 2005). These results demonstrate that the prefrontal regions are involved in the allocation of the increased attention required for a DT. However, many DT studies investigating cortical activity have not focused on the motor aspects of attention; while many experiments have used physical movement within their DTs, these have often involved fine movement of the hands in response to cognitive or perceptual tasks (Poldrack et al., 2005; Stelzel et al., 2006; Sigman and Dehaene, 2008; Tombu et al., 2011). Recent studies have attempted to address this issue by using volitional, self-initiated motor tasks, and a method that investigates cortical activity within motor regions. These experiments have resulted in mixed findings, with some demonstrating increased corticomotor activity (Hiraga et al., 2009; Van Impe et al., 2011), reduced activity (Master and Tremblay, 2009), and unchanged activity between DT and ST conditions (Sohn et al., 2005). These differences in results may be due to independent variables changing between DT studies. For example, Master and Tremblay (2009) use an additional task to divert attention away from the initial motor task, while in Van Impe et al. (2011) participants were instructed to simultaneously perform each task as well as possible to minimize the effect of DT interference. Here, the different type of DT tests between experiments may change the level of attention being directed to motor tasks, which may account for the changes in corticomotor activity.

Further limiting the application of DT results to accident prevention, few DT studies using motor tasks have compared older and younger participants. Van Impe et al. (2011) used fMRI during concurrent drawing, and mental arithmetic, to demonstrate higher activity within the supplementary motor area (SMA) for the young group during the DT condition (vs. ST condition), but not for the older group. These authors concluded that DT interference was not necessarily apparent in older group, despite the young participants being able to upregulate their SMA activity to a greater degree in response to the DT. Indeed, both groups were able to maintain DT performance, and the authors suggested the DT condition might not have been sufficiently challenging to cause participants to reach capacity of their cortical capabilities.

Two recent studies by Fujiyama et al. (2009, 2012), using TMS, also utilized a DT experiment involving motor tasks, comparing older and younger groups. Methods employed were similar between 2009 (mean age: young = 21.9 years; old = 66.7 years) and 2012 (mean age: young = 21.1 years; old = 69.1 years) studies, where single pulse TMS was used to measure the amplitude of motor evoked potentials (MEPs), and time of silent period (SP) duration from the motor “hot spot” of the forearm muscle (extensor carpi radialis) during ongoing ST (hand movement alone) and DT movement (hand and foot movement). Within TMS studies, the amplitude of the MEP with reflects the response of the corticospinal pathway after stimulation of the motor cortex, while the SP duration refers to an interruption of muscle activity caused by inhibition originating within the motor cortex (Wassermann et al., 2008). It is currently understood that this inhibition is mediated by of gamma-aminobutyric acid receptors (Wassermann et al., 2008).

Dual task movement in both studies by Fujiyama et al. (2009, 2012) involved concurrent hand and foot flexion and extension movements. Depending on different DT conditions included within the experiments, the hand and foot were required to be flexed and extended in the same, or opposite direction, in coordination with each other. Movements were also manipulated within experiments to include movement of the same, or opposite hand and foot. Fujiyama et al. (2009) revealed that younger adults had increased SP duration to certain DTs, while older groups did not, prompting the authors to suggest that older adults had a decreased ability to activate inhibitory function within the M1. Fujiyama et al. (2012) extended on the 2009 study by dividing and comparing DT coordination performance, and corticospinal inhibition, between “high” and “low” performing subgroups of older adults. Experiment one showed that conditions involving ipsilateral movements (using the same side of the body) of the hand and foot, and non-isodirectional movements (hand and foot movements in the opposite direction), were performed with less coordination (measured by the position of the hand and foot in relation to each other) than other conditions, with older adults having less coordination than younger adults during ipsilateral movements again. Within the purported most difficult condition of combined ipsilateral and non-isodirectional movements, TMS data showed that from the baseline ST condition, older adults’ SP duration decreased by 12.6% (*p* = 0.006), compared to an increase of 9% in SP duration (*p* = 0.11*)* for the younger group. The analysis of “high” and “low” performing older adults and SP duration was then conducted within this movement condition, revealing shorter SPs for the lower performing older subgroup than the higher performing older subgroup (*p* < 0.001). These results demonstrate a significant relationship between poorer DT motor performance in older adults and a reduction in inhibitory control within the M1. Control of motor performance is dependent on the activation of not only excitatory, but also inhibitory neurons within the motor cortex (Beck et al., 2008; Beck and Hallett, 2011), so interference caused by a concurrent task may result in underactivation of inhibitory neurons within the M1, evidenced within these TMS experiments by a reduction in SP duration.

Across the adult lifespan, not only do neurons within gray matter of the cortex shrink in size, but there is a loss of the myelin sheath insulating axons, resulting in a reduction in the propagation of impulses along the axon of the cell (Kramer and Willis, 2003). Importantly, this neurodegeneration does not affect the brain cortices uniformly; decreases in cortical volume associated with age are more prevalent in the prefrontal regions than other brain areas (Head et al., 2002). As already discussed, performance of a motor DT is dependent on prefrontal regions, and neurodegeneration of these regions with age suggests that the results from the TMS studies by Fujiyama et al. (2009, 2012) could be due to a reduced ability of the prefrontal regions of older adults to activate corticomotor inhibitory structures during a DT.

While there are no direct neural connections between prefrontal areas and the M1, it has been shown that in primates, prefrontal regions form dense connections to both ventral premotor (Dum and Strick, 2005) and dorsal premotor regions (Takahara et al., 2012). Therefore, to mediate attentional control of motor performance, it has been suggested that the premotor regions are anatomically suited to act as an intermediary between these two cortical regions (Picard and Strick, 2001). Supporting this view, Byblow et al. (2007) used two 50 mm figure-of-eight TMS coils to deliver a conditioning pulse to different areas of the premotor cortex, and a test pulse to the M1 over the “hot spot” of muscles of the forearm (extensor carpi radialis and flexor carpi radialis). Due to distinct modulation in activity within the M1 after dorsal premotor cortex conditioning, it was concluded that the connectivity between the dorsal premotor area and the M1 facilitates concurrent movement of the hand and foot. Marois et al. (2006), using concurrent tasks of speeded response selection and perceptual visibility, presented fMRI evidence suggesting that the dorsal premotor cortex is an important neural locus of these cortical limitations under DT conditions. Authors concluded that the flow of information from prefrontal regions during DT processing hits a “bottleneck” causing significant deficits in performance for these tasks. Thus, a reduction in the integrity of prefrontal structures with age could impair the activation of motor areas along this cortical pathway.

In addition to using TMS over the contralateral M1 in response to DT activity in experiment one, Fujiyama et al. (2012) conducted a second experiment where TMS was applied to the ipsilateral (right) M1 not responsible for the DT movement. ST and DT movements were the same as described in experiment one within Fujiyama et al. (2012), except that in addition, the left hand was tonically contracted, during right hand flexion/extension movement as part of the DT. This investigation was included to assess the contribution of *interhemispheric inhibition* (IHI) to changes in SP duration. Concurrent movements involving the right and left side of the body rely upon a balance of inhibition and facilitation between the left and right motor cortices via the corpus callosum (Fling et al., 2011). Importantly, the fiber tracts between left and right motor cortices are predominantly inhibitory rather than excitatory, with this inhibition between hemispheres thought to alleviate “motor overflow,” allowing right and left sides of the body to move independently of each other (Fling et al., 2011). Results demonstrated a significant shortening of the SP during right hand movement (and left hand tonic contraction), compared to the baseline condition in which only the left hand was tonically contracted. This indicates that IHI was active during the baseline condition to suppress unwanted right hand movement, but then “disinhibited” when the right hand movement was also necessary. Importantly, this shortening of the SP to the ipsilateral M1 was present in both older [7% reduction (*p* = 0.04)] and younger [3% reduction (*p* = 0.03)] groups, and no differences were seen in inhibition to the ipsilateral M1 in any other DT conditions within experiment two. This suggests that mechanisms of IHI during DT performance were not degraded within the older participants within this study.

Few other studies have used a DT involving motor tasks to measure inhibition of the M1. In those that have, a reduction in inhibition has also been demonstrated. Sohn et al. (2005) showed a clear decrease DT vs. ST, while Poston et al. (2012) demonstrated a decrease in inhibition for the DT vs. ST during movement initiation, but no change in during tonic contraction. The reduced SP in Sohn et al. (2005) was termed “disinhibition,” and it was proposed that this was advantageous in facilitating motor output to both muscles concurrently. Fujiyama et al. (2012) did not favor disinhibition as an explanation however, as performance was reduced with the DT movements, whereas in Sohn et al. (2005) DT performance was maintained. In Poston et al. (2012), MEP amplitude and SP duration both decreased during initiation of the DT movement. These results support the suggestion of a reduction in the ability to activate both excitatory and inhibitory networks within the motor cortex during attentionally demanding motor performance. However, this study did not include a comparison of older and younger adults, or of motor performance between conditions.

Motor performance, and corticomotor activity, may be dependent on a range of factors, such as task and movement type. For instance, it has been noted that older adults can outperform younger adults in interference tasks if their strategies are more appropriate to the task being tested (Worthy and Maddox, 2012). However, once the complexity of a task increases, older adults show increased strategy execution errors, particularly in an environment requiring extensive integration and weighing of information (Mata et al., 2012). This is in agreeance with the TMS results of Fujiyama et al. (2012), where older adults performance worsened on the most difficult DT condition. Therefore, age related declines in motor performance seem heavily dependent on task complexity, which is in accordance with the scope of this review, where DT articles were targeted that had used motor tasks that required responses that were voluntary and self initiated.

## Conclusion

This mini-review has demonstrated that while motor behavioral deficits occur in response to attentionally demanding motor performance, the cortical correlates of such a deficit have not been established. However, using a DT paradigm, recent TMS studies indicate that there may be a reduction in the ability of older adults to activate inhibitory networks within the M1, which are required for motor performance. A reduction in activation of corticomotor structures in older compared with younger adults is considered to be caused by the decline in the integrity of prefrontal regions with age (Head et al., 2002), which are rendered less able to allocate attention to both tasks simultaneously. Taking into account previous DT research investigating cortical activity, where responses have varied between studies, further research should be undertaken comparing cortical responses of older and younger adults during attentionally demanding motor performance.

## Conflict of Interest Statement

The authors declare that the research was conducted in the absence of any commercial or financial relationships that could be construed as a potential conflict of interest.
